# Osteoarthritis as an Enhanceropathy: Gene Regulation in Complex Musculoskeletal Disease

**DOI:** 10.1007/s11926-024-01142-z

**Published:** 2024-03-02

**Authors:** Jack B. Roberts, Sarah J. Rice

**Affiliations:** Skeletal Research Group, International Centre for Life, Biosciences Institute, Newcastle University, Newcastle Upon Tyne, NE1 3BZ UK

**Keywords:** Osteoarthritis, Gene regulation, GWAS, Enhanceropathy, Chromatin

## Abstract

**Purpose of Review:**

Osteoarthritis is a complex and highly polygenic disease. Over 100 reported osteoarthritis risk variants fall in non-coding regions of the genome, ostensibly conferring functional effects through the disruption of regulatory elements impacting target gene expression. In this review, we summarise the progress that has advanced our knowledge of gene enhancers both within the field of osteoarthritis and more broadly in complex diseases.

**Recent Findings:**

Advances in technologies such as ATAC-seq have facilitated our understanding of chromatin states in specific cell types, bolstering the interpretation of GWAS and the identification of effector genes. Their application to osteoarthritis research has revealed enhancers as the principal regulatory element driving disease-associated changes in gene expression. However, tissue-specific effects in gene regulatory mechanisms can contribute added complexity to biological interpretation.

**Summary:**

Understanding gene enhancers and their altered activity in specific cell and tissue types is the key to unlocking the genetic complexity of osteoarthritis. The use of single-cell technologies in osteoarthritis research is still in its infancy. However, such tools offer great promise in improving our functional interpretation of osteoarthritis GWAS and the identification of druggable targets. Large-scale collaborative efforts will be imperative to understand tissue and cell-type specific molecular mechanisms underlying enhancer function in disease.

## Osteoarthritis: A Complex Genetic Disease

Osteoarthritis is a degenerative disease of the articulating joint, most commonly the hip, knee, or hand. All joint tissues can be affected, resulting in synovial inflammation, subchondral bone thickening, osteophyte formation and ligament degeneration, yet the disease is conventionally hallmarked by cartilage degradation [1]. Osteoarthritis is common, impacting the lives of approximately 40% of adults over 70 [2], and is genetically complex. The proportion of osteoarthritis risk attributed to heritability has been estimated to be 22.5% at any joint site (14.7% for knee; 51.9% for hip) [3]. To date, over 100 independent single nucleotide variants (SNVs) significantly associated with osteoarthritis have been reported through genome-wide association studies (GWAS), emphasising the highly polygenic nature of this disease [4–6]. Therefore, these SNVs likely contribute to pathogenicity via modulation of enhancer activity, impacting the expression of a target (or disease effector) gene. Osteoarthritis risk SNVs individually exert modest effects (most with individual odds ratios < 1.5) [7] but the accumulation of multiple risk alleles can exceed the ‘liability threshold’ in which a tipping point is reached, subsequently leading to disease development and progression [8].

## ‘Enhanceropathy’ as a Disease Classification

Each cell within the human body shares an identical genome, yet individual populations exhibit strikingly distinct phenotypes to allow for their unique functional properties. The cellular plasticity that occurs throughout the life course is achieved by the stringent spatiotemporal expression of proteins. Underlying this expression are complex gene regulatory networks (GRNs) [9]. GRNs encompass the interplay between a gene and its regulators, including *cis-*regulatory elements (CREs), *trans*-acting transcription factors (TFs) and regulatory non-coding RNAs (ncRNAs) [10].

CREs are genomic regions that can be categorised as promoters (proximal to a gene’s transcriptional start site), repressors, insulators (which define topologically associating domains (TADs) within the 3D genome), or enhancers [11]. Enhancers typically reside within non-coding (intergenic and intronic) regions and consist of numerous transcription factor (TF) binding sites that facilitate gene regulation [12]. They can be up- or downstream of their target gene, within a gene body, or physically distal. Multiple models have been proposed for the mechanism of physical interaction between enhancers and promoters, yet the consensus is that enhancers are brought into physical proximity with a promoter through chromatin looping, amplifying the recruitment of RNA Pol II for transcription [13].

**Fig. 1:**
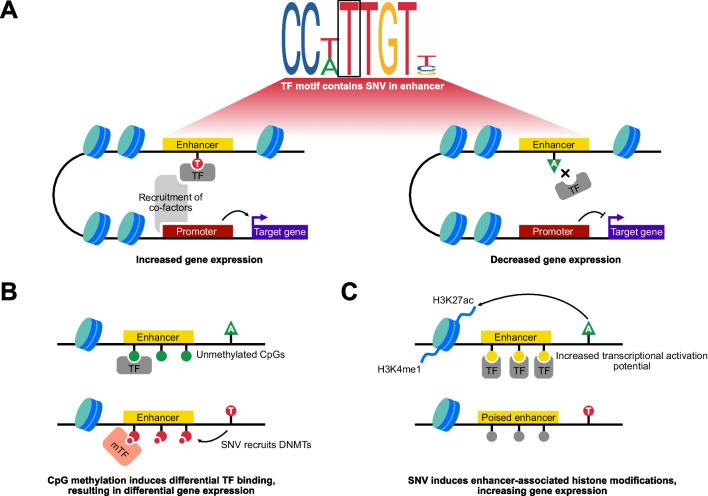
SNVs can directly or indirectly affect enhancer activity. **A** The presence of a single nucleotide variant (SNV) within a transcription factor (TF) binding motif alters TF binding affinity within an enhancer region. Left, TF binds in the presence of the T allele, resulting in the recruitment of co-factors and interaction with the promoter of a target gene. This results in increased target gene expression. Right, the A allele within the binding motif of the TF reduces TF binding, decreasing enhancer activity and downstream gene expression. **B** SNVs modulate proximal CpG methylation status, leading to differential TF binding and enhancer activity. Top, the A allele has no effect on proximal CpG methylation status; therefore, TFs that preferentially bind unmethylated CpGs bind the enhancer and regulate target gene expression. Bottom, the T allele recruits DNA methyltransferase enzymes (DNMTs) that increase proximal CpG methylation status; therefore, TFs that preferentially bind methylated CpGs (mTF) bind the enhancer and regulate target gene expression. This effect can operate in reverse or lead to TF competition for binding site occupancy. **C** SNVs affect the expression of genes encoding histone modifiers. This results in altered patterns of histone modifications and enhancer activity. Top, the A allele induces enhancer-associated histone modifications, including histone 3 lysine 27 acetylation (H3K27ac) and histone 3 lysine 4 mono-methylation (H3K4me1), activating enhancer activity and increasing transcriptional activation potential of target genes. Bottom, the T allele does not affect histones proximal to the poised enhancer, leaving it inactivated and reducing the transcriptional activation potential of target genes

In 2014, Smith and Shilatifard coined the term ‘enhanceropathies’ as a novel classification of disease [14•]. Such diseases encompass three distinct mechanisms by which altered enhancer function can underlie pathology: disruptions to enhancer-promoter interactions through chromosomal deletions or rearrangements (including β-thalassemia and Burkitt’s lymphoma [15, 16]), rare mutations within genes encoding regulators of enhancer activity (including *P300* and *KMT2D* [17, 18]), and common polymorphism within enhancer sequences [19]. Human SNVs falling within gene enhancer regions can alter the binding affinity of transcription factors, subsequently leading to changes in target gene expression (Fig. 1A) and contributing to phenotypic variation, including disease.

## Epigenetic Influences upon Enhancer Function

The methylation of DNA (DNAm) at cytosine-guanine dinucleotides (CpGs) is the most widely studied epigenetic mark [20]. DNAm is intrinsically linked to transcriptional regulation, commonly gene repression, by preventing the binding of transcriptional activators to promoter regions and through the recruitment of repressive methyl-binding proteins. However, the relationship between DNAm and gene expression is far from straightforward, with gene body methylation often correlating with active transcription [21]. Generally, it is considered that DNAm within enhancers is repressive to the expression of a target gene [22], with the ‘active’ enhancer histone modification, H3K27ac, negatively correlating with DNAm levels in multiple cell and tissue types [23]. However, traditional bulk analyses of enhancer states often fail to directly correlate methylation state with gene expression, potentially due to cell-type heterogeneity [24]. This has been confirmed most recently by Kreibich et al*.* who employed single molecule footprinting in mouse embryonic stem cells to demonstrate that CpGs with negative DNAm-chromatin accessibility (CA) correlations are most frequently located centrally within enhancers, where CA is the highest [25••]. They further identified that negative DNAm-CA correlations within enhancers are cell-type specific but, where such a relationship exists, the DNAm *can* directly modulate the recruitment of TFs to the CRE. This relationship is depicted in Fig. 1B.

Whilst environmental factors are traditionally considered to be the principal factor governing changes to the methylome, a considerable proportion (10–20%) of DNAm is regulated *in* *cis* by genotype at a proximal SNV [26–29]. Co-localisation analysis, which tests whether a shared variant has a causal impact on both disease risk and DNAm, has identified that ~ 25% of osteoarthritis SNVs co-localise with methylation Quantitative Trait Loci (mQTLs) in human adult articular cartilage [4]. This interplay between the genome and epigenome supports an important role for CpG methylation in the molecular mechanisms underlying osteoarthritis.

**Fig. 2:**
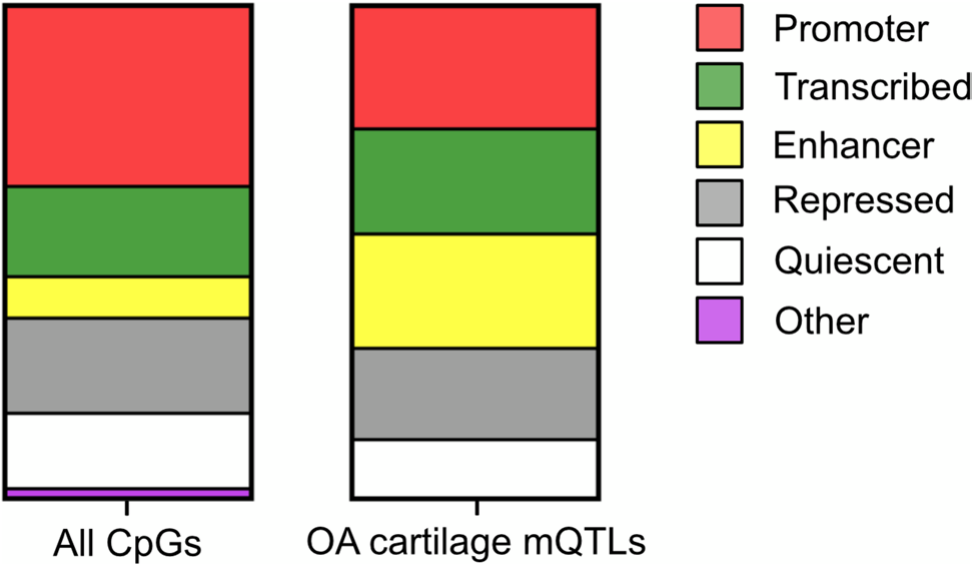
Osteoarthritis cartilage mQTLs are enriched in chondrocyte enhancer regions. Intersection of CpGs on the Illumina Infinium HumanMethylation450 BeadChip array (left) and the 108 reported osteoarthritis cartilage mQTLs (right) with chromatin state data from the Roadmap Epigenomics Project in MSC-derived chondrocytes (E049) reveals enrichment for cartilage mQTLs in enhancer-annotated regions (*P* < 0.0001) and depletion in promoter-annotated regions (*P* = 0.0123). No significant difference was identified for transcribed (*P* = 0.4549), repressed (*P* = 0.9035), quiescent (*P* = 0.4223), or other regions (*P* = 0.2869). Intersection was performed using ‘bedtools Intersect intervals’ within Galaxy. Statistical test: Fisher’s exact test (GraphPad Prism 10)

The integration of epigenetic datasets with GWAS signals has facilitated the statistical fine mapping of SNVs and the prioritisation of effector genes [5••]. This includes the identification of mQTLs falling within annotated CREs in relevant cell types. Of the 108 reported CpGs comprising cartilage osteoarthritis mQTLs (OA-mQTLs) [30–34], 23.1% fall within annotated chondrocyte enhancers and 25% within promoters (Fig. 2). This corresponds to a significant enrichment of osteoarthritis mQTLs in articular chondrocyte enhancers (*P* < 0.0001) and depletion in  promoters (*P* = 0.0123). The distribution of CpG sites on the Infinium HumanMethylation450 BeadChip array is heavily weighted towards promoters rather than enhancers [35]. This suggests that the current figures of OA-mQTLs may be an underrepresentation of the true number. Increasing epigenome coverage offered by the next generation of arrays including the HumanMethylationEPIC (EPIC) should provide more accurate estimates. This is supported by a recent mQTL analysis of DNA from the infrapatellar fat pad, which utilised the EPIC array and identified co-localisation with 44% of tested osteoarthritis SNVs [36]. The identification of OA-mQTLs is integral in the prioritisation of putative disease enhancers.

In recent years, several studies have used epigenetic editing to *functionally* link OA-mQTLs and their enhancers to effector genes. These have included the expression of deactivated Cas9 (dCas9) fused to the epigenetic modulators DNMT3a, a de novo DNA methyltransferase, and the de-methylating enzyme TET1, precisely editing chondrocyte DNAm in vitro. This functional fine-mapping approach has identified mechanistic links between mQTLs and further confirmed *COLGALT2* [37–39*TGFB1* [40], *RWDD2B* [41] and, most recently, *WWP2* [42] as osteoarthritis effector genes. *WWP2* encodes an E3 ubiquitin ligase with multiple isoforms known to target different Smad signalling proteins [43]. Here, the osteoarthritis risk allele (G) at rs34195470 was shown to correlate with increased DNAm at 14 CpGs within the gene body in chondrocytes isolated from osteoarthritis cartilage [42]. Using dCas9-DNMT3a to increase DNAm levels at these CpGs in TC28a2 immortalised chondrocytes, effectively recapitulating the observed mQTL effect, resulted in increased expression of full-length and N-terminus *WWP2*, confirming these isoforms as targets of osteoarthritis risk. This supported earlier reports of allelic expression imbalance of *WWP2* in articular cartilage [44, 45] and uncovered the functional molecular mechanism underlying an osteoarthritis effector gene.

As both the DNA methylome and GRNs are heavily dependent upon cellular context, it is vital to investigate such effects in disease-relevant cell lines and tissues. This further complicates osteoarthritis research, beyond the heterogeneity of tissues themselves, as choosing the ‘correct’ tissue is not always straightforward. Increasingly, studies of this disease are expanding to include non-cartilaginous tissues. Recently, co-localisation of osteoarthritis risk signals and mQTLs has been conducted in other osteoarthritis-relevant tissue types, revealing significant mQTLs in both synovium [46••] and fat pad [36], a proportion of which appear to exert tissue-specific effects. We discuss the tissue specificity of osteoarthritis enhancers in more detail below.

## Osteoarthritis Risk SNVs and Chromatin Remodelling Proteins

Enhanceropathies encompass pathologies that result in direct inhibition of TF binding to enhancer regions and, additionally, those which affect chromatin state and enhancer accessibility. Several osteoarthritis-associated SNVs map to genes encoding post-translational modifiers of histone proteins, including the histone methyltransferase gene *DOT1L* and the histone acetylation and de-ubiquitinase gene *SUPT3H* [47, 48]. Loss of DOT1L-mediated methylation of histone 3 lysine 79 (H3K79me) following the addition of the small molecule S-adenosyl methionine competitive inhibitor EPZ-5676 in human primary articular chondrocytes has been shown to reduce the expression of chondrocyte markers *COL2A1* and *ACAN*. Moreover, intra-articular injection of EPZ-5676 into the knee of adult mice triggered cartilage loss marked by histological staining [49]. To the best of our knowledge, no functional data has been reported describing the role of SUPT3H in cartilage. However, allelic expression imbalance (AEI) studies using nucleic acids from human articular chondrocytes have identified a risk allele correlating with increased *SUPT3H* expression in synovium, cartilage and trabecular bone samples derived from patients undergoing arthroplasty for primary osteoarthritis [50]. Together, these results are suggestive that dysregulation of histone modification proteins, and a subsequent change in chromatin accessibility and enhancer function, can contribute to osteoarthritis pathogenesis (Fig. 1C). Further investigations are required to understand the mechanisms underlying such dysregulation, and characterise the functional impact upon the epigenome.

## Chromatin State at Chondrocyte Enhancers in Osteoarthritis

Over the last decade, multiple technologies have been developed and successfully applied to identify tissue-specific gene enhancers. The main techniques along with their respective advantages and limitations are outlined in Table 1. These technologies quantify a range of parameters to define chromatin state ranging from CA, long-range interactions (LRI), and post-translational histone modifications (PTMs). Together, they enable the designation of active enhancer elements and their target genes in disease-relevant cell types. Many public databases have made such datasets available across multiple cell and tissue types and are summarised in Table 2. The availability of epigenomic datasets has enabled the prioritisation of enhancer elements for functional follow-up studies and the identification of osteoarthritis effector genes [4].

**Table 1:** Technologies used to investigate enhancer function

Approach	Brief description	Advantages	Disadvantages	Ref
**ATAC-Seq** (Assay for Transposase Accessible Chromatin with high-throughput Sequencing)	Maps regions of open, accessible chromatin genome wide. Tn5 transposase cuts DNA and ligates adapters for high-throughput sequencing at regions of increased accessibility (i.e. nucleosome-sparse, open chromatin regions)	• Can detect open chromatin • Low cell numbers required (~ 50,000) • The protocol can be performed in hours • No need for sonication, phenol–chloroform extraction, or antibodies	• Requires isolation of single nuclei • Over-digestion: more reads mapping to inaccessible genomic regions • Under-digestion: high molecular weight fragments, which are more difficult to sequence	[51]
**FAIRE-Seq** (Formaldehyde-Assisted Isolation of Regulatory Elements with Sequencing)	Isolates and identifies nucleosome-depleted regions of the genome. Chromatin is crosslinked with formaldehyde and then sheared by sonication and extracted using phenol–chloroform phase separation prior to sequencing	• Does not require antibodies or enzymes • No requirement to isolate single cells or nuclei isolation • Simple protocol, highly reproducible	• High cell numbers required (10^5^–10^6^)	[52]
**DNase-Seq** (DNase I hypersensitivity sites with sequencing)	Maps regions of open, accessible chromatin genome-wide at DNase I hypersensitivity sites (DHS). Accessible regions of the genome are subject to cleavage by DNase I. These regions typically map to regulatory elements such as promoters or enhancers	• Has greater sensitivity at promoters than FAIRE-Seq • Can detect open chromatin	• High cell numbers required (10^5^–10^6^) • DNase I is sequence-specific: hypersensitive sites may not represent entire genome • Multiple purification steps lead to DNA loss and reduced sensitivity	[53]
**Chromatin conformation capture** (Hi-C, 3C, Capture-C)	A set of techniques to identify interactions between chromosomal regions. DNA–protein complexes are crosslinked with formaldehyde, and then, the sample is fragmented and digested with restriction enzymes. Resultant DNA fragments are amplified and sequenced	• High-throughput detection of long-range chromatin interactions	• Requires large amounts of starting material	[54–56]
**ChIA-PET** (Chromatin Interaction Analysis by Paired-End Tag sequencing)	Immunoprecipitation to map long-range chromatin interactions. DNA–protein complexes are crosslinked and fragmented, and then, specific antibodies are used for immunoprecipitation. Deep sequencing provides base-pair resolution of ligated fragments, allowing the identification of chromatin interactions	• Can detect both long-range and short-range chromatin interactions	• Requires a large number of cells (> 100 million) • Linkers can self-ligate, leading to ambiguity in true interactions	[57]
**ChIP-Seq** (Chromatin ImmunoPrecipitation with high throughput Sequencing)	Can be used to detect histone modifications or TF binding. Identifies specific location of proteins that are directly or indirectly bound to genomic DNA	• Defines TFBS • Histone modifications can inform chromatin state datasets using publicly available modelling (e.g. ROADMAP)	• Large cell numbers required • Sub-optimal signal to noise ratio	[58]
**CUT&RUN** (Cleavage Under Targets and Release Using Nuclease)	Detects histone modifications and TF binding to DNA by selectively cleaving antibody-tagged chromatin with a micrococcal nuclease (MNase), which can then be analysed by next-generation sequencing	• Requires fewer cells and is more economical than ChIP-Seq • Can define histone modifications and TFBS genome wide • Improved signal-to-noise ratio compared to ChIP-Seq	• Antibodies previously validated for use with ChIP-Seq may not be suitable for CUT&RUN and require validation	[59]

**Table 2:** Summary of the existing databases providing useable genomic and epigenomic data relevant to enhancer biology

Source	Data type(s)	Description	Weblink
3D Genome Browser	• ChIA-PET • Hi-C	Visualisation of chromatin interactions for genomic regions of interest. 6 tissue types are available for ChIA-PET; 45 tissue types are available for Hi-C	http://3dgenome.fsm.northwestern.edu/
ENCODE	• Histone and chromatin-associated protein ChIP-Seq • ATAC-Seq • DNase-Seq • ChIA-Pet • Hi-C • DNAm array	An encyclopaedia of DNA elements, including ROADMAP data, covering 7.9% and 3.4% of human and mouse candidate *cis-*regulatory elements, respectively	https://www.encodeproject.org/
FANTOM5	• Cap Analysis of Gene Expression (CAGE)	An atlas of promoters and enhancers created by CAGE peaks, enriched by histone ChIP-Seq and DNase-Seq data	https://fantom.gsc.riken.jp/5/
JASPAR	• Transcription factor binding sites	A manually curated transcription factor binding motif database. Validated motifs (identified by ChIP-Seq) and unvalidated motifs are included. Binding motifs are available as UCSC tracks as ‘JASPAR Core’	https://jaspar.elixir.no
ReMap	• ChIP-Seq	A collection of 2829 transcriptional regulator (TFs, co-activators and chromatin regulators) ChIP-Seq datasets across 346 cell types	http://remap.cisreg.eu/
ROADMAP	• Histone ChIP-Seq • DNase-Seq	A repository of 127 histone ChIP-Seq reference epigenomes, including osteoarthritis-relevant cell types Chromatin state learning models can be applied to own datasets to segment genome into functional and regulatory elements such as enhancers DNase-Seq data is also available	https://egg2.wustl.edu/roadmap/web_portal/
WashU Epigenome Browser	• ChIA-PET • Hi-C	Supports navigation and visualisation of epigenomic data	http://epigenomegateway.wustl.edu

### Histone Post-Translational Modifications (PTMs)

Histone PTMs provide valuable information on chromatin state. Typically, histone 3 lysine 4 mono-methylation (H3K4me1) and H3K27 acetylation (H3K27ac) are associated with enhancer activity. Other histone marks are associated with transcriptionally repressed regions (H3K27 tri-methylation, me3), active promoters (H3K4me3) and actively transcribed regions (H3K36me3). Performing chromatin immunoprecipitation with high-throughput sequencing (ChIP-Seq) on histone modifications has facilitated the epigenome-wide annotation of regulatory elements within different cell types, defined by specific combinations of histone marks. This provides a useful tool for prioritising enhancer regions in specific cell types (Table 1). Several large-scale projects have defined histone PTMs across many cell types and provide a useful resource for investigators, including ENCODE, FANTOM and Roadmap (Table 2) [60–62, 62

In 2020, Cheung et al. performed histone ChIP-Seq on hMSCs cultured in monolayer or differentiated into chondrocytes [63]. Classification of histone ChIP-Seq data using a 16-state chromatin model showed a high degree of similarity of enhancer regions (marked by H3K4me1 and H3K27ac modifications) between terminally differentiated hMSCs and Roadmap E049 chondrocytes. Integration with epigenome-wide DNAm array data identified that CpGs that became demethylated during chondrogenesis were overrepresented in enhancer regions. To assess the functional role of DNAm at these putative enhancers, six regions encompassing the demethylated sequences were cloned into luciferase reporter vectors and, in all cases, the unmethylated enhancer sequence demonstrated increased reporter activity in the SW1353 chondrosarcoma cell line when compared to methylated vectors. These functional validation studies suggest that DNAm modulates TF binding and chondrocyte enhancer activity at these sites (Fig. 1B).

The utility of integrating histone ChIP-Seq data with other epigenetic datasets is an effective strategy towards enhancer identification. The availability of large public datasets (Table 2) provides a valuable resource for investigators to apply to their own studies. Future studies aiming to characterise PTMs in primary cells, which can be challenging to collect in sufficient numbers required for ChIP-Seq, may choose to opt for CUT&RUN (Cleavage Under Targets and Release Under Nuclease) [59] (Table 1). Using CUT&RUN, which requires as few as 10,000 cells per assay, Sarkar et al. have recently investigated binding sites of the TF STAT3 in human foetal, adult, and osteoarthritis chondrocytes [64].

### Chromatin Accessibility (CA)

Open, accessible chromatin facilitates the binding of TFs that modulate gene expression. Therefore, measuring CA in relevant cell types can provide valuable insight into cell type-specific CREs [65]. Historically, DNase I Hypersensitivity Site (DHS) [66] with sequencing (DNase-Seq), which identifies nucleosome-depleted regions of the genome that are accessible for cleavage by DNase I, and Formaldehyde-Assisted Isolation of Regulatory Elements with Sequencing (FAIRE-Seq) [67], which utilises phase separation of crosslinked protein-DNA structures and high-throughput sequencing, have been used to identify accessible chromatin (Table 1). However, these technologies are limited by the requirement for large cell numbers which can be difficult to acquire in matrix-dense, hypocellular tissues such as bone and cartilage. More recently, the Assay for Transposase Accessible Chromatin with Sequencing (ATAC-Seq) was developed. This technology employs Tn5 transposase to ‘tagment’ accessible DNA via cleavage and tagging with sequencing adaptors, facilitating the detection of open chromatin regions whilst requiring as little as 50,000 cells (Table 1) [68]. To date, five osteoarthritis-relevant ATAC-Seq studies have been reported.

In 2018, Liu et al*.* mapped chromatin accessibility in human articular chondrocytes (hACs) derived from eight Japanese primary osteoarthritis patients undergoing knee arthroplasty [69]. They identified 109,215 accessible chromatin regions, of which 71% reside within enhancers marked by Roadmap DHS annotations, cross-validating ATAC-Seq against the more established DNase-Seq. They intersected the peaks with the physical location of osteoarthritis-associated SNVs and found that 68% fell within accessible chromatin regions, again emphasising the role of these regulatory elements in osteoarthritis gene dysregulation.

The role of CA in driving differential gene expression in osteoarthritis was further supported by an independent ATAC-seq study in 2021. Barter et al*.* demonstrated that stimulation of the chondrosarcoma cell line SW1353 with proinflammatory cytokine Interleukin-1 (IL-1) resulted in 241 significant differentially accessible regions (DARs), which were enriched in Roadmap chondrocyte enhancers [70]. Conversely, the changes were underrepresented in promoter regions, suggesting that the disruption of GRNs within the joint in response to inflammatory stimuli is predominantly driven by enhancers. Furthermore, the authors functionally validated these regions in driving the inflammatory response using CRISPR-Cas9 to delete an IL-1-induced open chromatin region within *MMP13*, encoding matrix metalloproteinase-13 (a well-characterised initiator of cartilage catabolism) from the genome of SW1353 immortalised chondrosarcoma cells. Deletion of this gene enhancer resulted in an attenuated upregulation of *MMP13* following IL-1 stimulation.

To date, the most comprehensive analysis of a gene enhancer associated with osteoarthritis pathogenesis [71••] was published in 2020. Disease-associated SNVs mapping to *GDF5*, encoding growth differentiation factor 5, a bone morphogenic protein with known roles in mammalian knee development [72], were intersected with embryonic mouse and human knee ATAC-Seq peaks to prioritise putative causal variants. Richard et al*.* identified the presence of rs6060369 within a common knee open chromatin region in mice and humans [71••], the deletion of which resulted in reduced *GDF5* expression in the chondrocyte cell line TC28a2. Murine studies of the CRE further demonstrated that deletion of the region resulted in morphological changes to condyle curvature and width and led to the development of osteoarthritis in aged mice. Computational modelling predicted that rs6060369 occupied and disrupted the TF binding site for pituitary homeobox-1 (PITX1), a critical TF for knee development [73], which was functionally validated using ChIP-Seq, supportive of the enhanceropathy model depicted in Fig. 1A. This study was the first to demonstrate that an osteoarthritis-associated enhancer variant controlling early development of the human knee joint can predispose humans to osteoarthritis in later, post-reproductive life: a phenomenon known as antagonistic pleiotropy [74].

To further understand the developmental origins of the functional gene dysregulation that contributes to osteoarthritis and temporal changes in chromatin accessibility in cartilage, our laboratory performed ATAC-Seq on 12 human foetal cartilage samples taken from the proximal (hip) and distal (knee) ends of developing long bones and 10 osteoarthritis cartilage samples from patients undergoing arthroplasty at hip and knee joint sites [32•]. Significant DARs (113,887 hip and 121,050 knee) were identified between foetal and osteoarthritis cartilage. Once more, these regions showed significant enrichment of enhancer annotations, indicating that changes in gene expression are driven by altered enhancer function (rather than promoters). Interestingly, 36 osteoarthritis-associated SNVs overlapped with ATAC-Seq peaks uniquely in foetal cartilage (*n* = 16) or osteoarthritis cartilage (*n* = 20), suggesting that genetic determinants of osteoarthritis risk may function at specific stages of the life course. Future functional studies and fine-mapping of risk loci to target genes must consider tissues taken throughout the life course, post-development, yet before disease initiation.

Understanding disease-specific changes to enhancer accessibility contributes to our understanding of pathology. In 2023, Wang et al*.* applied ATAC-Seq to identify DARs between primary chondrocytes taken from patients with osteoarthritis (*n* = 2) or Kashin-Beck disease (KMD, *n* = 2) to understand specific differences in these cartilage-degrading diseases. Of the 51,900 accessible chromatin peaks identified for osteoarthritis chondrocytes, 14,541 were not present in KMD chondrocytes. These uniquely accessible chromatin regions may therefore provide insight into osteoarthritis-specific enhancer dysregulation and warrant further investigation.

### Long Range Chromatin Interactions (LRI)

The spatial organisation of the non-linear genome provides important context to regulatory elements that act at physically distal regions. Chromosome conformation technologies are therefore valuable tools to determine targets of enhancer activity. Chromatin conformation capture technologies (including Capture Hi-C) allow high-throughput detection of LRIs following DNA–protein crosslinking and base-pair resolution sequencing (Table 1) [75]. These LRI maps depicting enhancer-promoter interactions can provide insights into the molecular mechanisms by which SNVs drive susceptibility to common, complex diseases [76]. For example, the application of Capture Hi-C data in human mesenchymal stem cells (hMSCs) has identified that the thumb osteoarthritis SNV rs11588850, which resides within the gene body of *SNAP47*, physically interacts with the 200 kb-upstream transcription start site (TSS) of *WNT9A*. The *WNT9A* gene is differentially expressed between high and low-grade osteoarthritic cartilage and known to play a key role in joint formation [77, 78].

Additional studies have applied chromatin conformation data *in silico* to prioritise osteoarthritis risk SNVs and effector genes. Using existing Chromatin Interaction Analysis by Paired-End Tag sequencing (ChIA-PET) data (Table 1), Kehayova et al. identified physical interactions between the locations of osteoarthritis mQTL CpGs identified in adult cartilage tissue and the 3′ untranslated region of *COLGALT2*, subsequently confirming this as an osteoarthritis effector gene through functional analysis [37]. Similarly, LRIs were identified between the TSSs of *TMEM129* and *SLBP* and an osteoarthritis-associated differentially methylated region, with functional studies confirming a regulatory role of this putative enhancer in modulating *TMEM129* expression in chondrocytes [79].

## Tissue-Specific Enhancers Within the Osteoarthritic Joint

Historically, investigations into osteoarthritis aetiology at the molecular level have been cartilage-centric, yet progressively the paradigm is shifting to consider osteoarthritis as a disease affecting the whole joint [1]. Transcriptomic and epigenomic studies increasingly include non-cartilaginous joint tissues, such as synovium [46, 80], infrapatellar fat pad [36] and subchondral trabecular bone [81, 82].

Direct comparisons of enhancer methylation status at the sites of known OA-mQTLs have identified one notable example of opposing allelic impacts upon DNAm measured in distinct joint tissue environments [39••]*.* At this locus, harbouring the gene *COLGALT2* and marked by osteoarthritis risk SNV rs11583641, 8 CpGs cluster within a 500-bp region of an intronic enhancer. In osteoarthritis cartilage, 3/8 CpGs exhibited significant mQTLs, with the major (risk) allele correlating with decreased levels of DNAm. Functional expression studies involving CRISPR-Cas9 deletion of the region and precision editing of the methylome at this site confirmed *COLGALT2* as the target gene, with a decrease in methylation corresponding with an increase in gene expression [37]. Interestingly, this epigenetic effect was much greater in human foetal cartilage, and the chromatin at the enhancer was significantly more accessible, indicating that the conferred overexpression of the protein in cartilage in those carrying the risk allele is also active during skeletal development [83]. The discussion of the role of enhancers during cartilage development, and how this contributes to osteoarthritis in later life, was recently intricately described [84] and so has been excluded from the scope of this review.

Analysis of the identified OA-mQTLs within adult osteoarthritis synovium revealed that at *all 8* enhancer CpGs, the risk allele correlated with significantly increased DNAm, and a subsequent decrease in gene expression, between which a functional link was again confirmed through epigenome editing [39••]. This is an example of biological pleiotropy, in which the impact of a risk variant (or haplotype) produces a different phenotypic outcome between two tissues. Such effects are known and already add substantial complexity to the pharmacological targeting of pathways resulting from GWAS studies. It is estimated that ~ 30% of GWAS SNVs exhibit pleiotropic effects (associating with multiple traits or diseases) which map to > 60% of genes [85]. However, such antagonistic effects are seemingly rare within multiple tissues of the same organ, which have the potential to contribute to the *same* disease. To date, relatively few studies have directly contrasted such effects between tissues of the articulating joint, and amongst those that have, only a small number of all known osteoarthritis risk loci have been included [41, 46, 50, 86]. On an epigenome-wide scale (considering *all* SNV-CpG correlations), Kreitmaier et al*.* identified just 33 mQTL pairs demonstrating an antagonistic effect between osteoarthritis knee cartilage and synovium (0.02%) [34]. The extent to which such biological pleiotropy within the joint could impact future pharmacological targeting of osteoarthritis remains unclear. Rigorous molecular investigations into the impact of SNVs upon target genes, coupled with biological studies into the encoded protein function, within the context of multiple joint tissues are essential to inform pre-clinical drug development studies.

Finally, the consideration of cartilage as a heterogeneous tissue must not be overlooked. Mature articular cartilage has long been considered to consist of a single cell type: the articular chondrocyte. Whilst this central dogma still stands, the advent of single-cell (sc) technologies has revealed and defined subsets of articular chondrocytes [87] within both diseased and healthy tissue [88] at the transcriptome level. To date, scATAC and scMethyl-seq have not been applied in human chondrocytes, yet bulk sequencing technologies have paved the way for the identification of disease-driving chondrocyte subsets within cartilage and the identification of subset-specific enhancers [89]. It is wholly possible that in cartilage, as has been described in other tissues, bulk epigenomic investigations can mask correlations between the epigenome and transcriptome.

## SNV to Gene Studies: The Missing Link and Future Directions

In keeping with the ‘liability threshold’ model, an individual who inherits sufficient osteoarthritis risk alleles is likely to exhibit aberrant enhancer function and dysregulation of essential genes for normal joint development and function, resulting in osteoarthritis. However, functional interpretation of osteoarthritis risk SNVs is impeded by several factors: they often reside within linkage disequilibrium (LD) blocks, meaning reported variants (the array tag SNVs) are likely a proxy for the causal variant; SNVs exert tissue-specific effects, i.e., they may regulate a target gene in one tissue whilst having no effect on gene expression in another or even have opposing effects in distinct tissues; and the three-dimensional structure of chromatin may result in SNVs regulating genes that are physically close but linearly distant. Unravelling these mechanisms therefore relies on researchers being able to characterise regulatory elements in disease-relevant tissue types, combining chromatin organisation and structure with epigenetic marks such as DNAm and histone modifications.

Effector genes of complex traits, including osteoarthritis, have been prioritised through powerful statistical fine-mapping approaches, including the co-localisation of causal candidate SNVs with expression QTL (eQTL) from datasets such as GTEx. However, evidence suggests that less than half of GWAS signals co-localise with eQTLs [90]. One further limitation to this approach is the tissue-specificity of eQTLs (as also observed with mQTLs), creating a hindrance to conclusive results in diseases such as osteoarthritis, where there is a lack of disease-relevant datasets. A recent study, applying scRNA-seq in circulating immune cells, found no evidence to support that cell-type QTL specificity arises from differences in gene expression, or from low statistical power, indicating that transcription factor expression and/or binding site accessibility (within enhancers) drive such effects [91]. In addition to the contribution of cellular context to the co-localisation of eQTLs with GWAS signals, it has been reported that a limitation of this overlap is in part because GWAS and eQTL studies are powered to identify different types of SNV. This report, from Mostafavi et al*.*, demonstrated using GWAS analysis of the UK Biobank (in 44 complex traits), and GTEx eQTL data (in 38 tissues) that GWAS hits fall within regions of high evolutionary constraint, and their effector genes have large, complex regulatory elements, enriched for functional annotation, unlike eQTLs [92•]. Whilst similar biases are predicted in the discovery of other molecular QTLs, it has been shown that epigenetic QTLs are more highly enriched for disease heritability.

Across the field of complex disease research, including osteoarthritis, a multifaceted interdisciplinary approach is required to identify the target genes of enhanceropathies. The integration of multiple lines of larger genetic, epigenetic, and transcriptomic datasets, generated in relevant tissues throughout the life course, must be combined with powerful functional tools such as Cas9 (epi)genome editing and massively parallel reporter assays [93]. Such endeavours will only be bolstered by recent advances in single-cell technologies yet still require the global collaborative efforts of osteoarthritis researchers to combine resources and expertise.
